# Analysis of Group ICA-Based Connectivity Measures from fMRI: Application to Alzheimer's Disease

**DOI:** 10.1371/journal.pone.0049340

**Published:** 2012-11-30

**Authors:** Shanshan Li, Ani Eloyan, Suresh Joel, Stewart Mostofsky, James Pekar, Susan Spear Bassett, Brian Caffo

**Affiliations:** 1 Department of Biostatistics, Johns Hopkins University, Baltimore, Maryland, United States of America; 2 Department of Radiology, Johns Hopkins University, Baltimore, Maryland, United States of America; 3 Kennedy Krieger Institute, Baltimore, Maryland, United States of America; 4 Department of Neurology, Johns Hopkins University, Baltimore, Maryland, United States of America; 5 Department of Psychiatry and Behavioral Sciences, Johns Hopkins University, Baltimore, Maryland, United States of America; Vanderbilt University, United States of America

## Abstract

Functional magnetic resonance imaging (fMRI) is a powerful tool for the in vivo study of the pathophysiology of brain disorders and disease. In this manuscript, we propose an analysis stream for fMRI functional connectivity data and apply it to a novel study of Alzheimer's disease. In the first stage, spatial independent component analysis is applied to group fMRI data to obtain common brain networks (spatial maps) and subject-specific mixing matrices (time courses). In the second stage, functional principal component analysis is utilized to decompose the mixing matrices into population-level eigenvectors and subject-specific loadings. Inference is performed using permutation-based exact logistic regression for matched pairs data. The method is applied to a novel fMRI study of Alzheimer's disease risk under a verbal paired associates task. We found empirical evidence of alternative ICA-based metrics of connectivity when comparing subjects evidencing mild cognitive impairment relative to carefully matched controls.

## Introduction

Functional magnetic resonance imaging (fMRI) is a driving force in the field of brain mapping and cognitive neuroscience. It has been used as a noninvasive tool for describing and quantifying normal and abnormal brain function through the theory of neuro-vascular coupling. Functional connectivity is the study of correlations in measured neural signals [1]. Our study is motivated by the fact that differences in functional connectivity have been proposed to be associated with Alzheimer's disease [2]–[4]. In this manuscript, our objective is to examine whether independent component analysis (ICA)-based analysis of task-related fMRI presents evidence of differences in connectivity between subjects with mildly cognitive impairment and carefully matched controls.

We approach our study of fMRI by simultaneously analyzing all voxels. This is in contrast to regional or seed-based approaches [3], [5], [6] that restrict attention to carefully chosen locations. Such approaches require strong assumptions on the choice of seeds or parcellation used to define regions. Hence voxel-wise approaches are important complementary procedures. However, given the volume of voxels under study, flexible yet parsimonious models are required.

Independent component analysis is a factor-analytic approach that has been frequently utilized for the analysis of functional neuroimaging data [7]–[10]. We focus entirely on noise-free group spatial independent component analysis. To summarize, the model is noise free by not assuming a residual error term. It is spatial, by assuming that spatial independent components (ICs) drawn from statistically independent distributions mix over time via fixed effects to be estimated. And it is standard group ICA, achieving parsimony by assuming common spatial ICs across subjects yet different temporal mixing matrices. These assumptions are the de facto starting point for group factor analysis investigations of fMRI data.

Functional principal component analysis (FPCA) is a common method to capture the main directions of variation and dimension reduction in a collection of functions [11]–[13]. We use FPCA to identify the population-level eigenvectors that characterize the geometric directions of variation of the time courses acquired from ICA. FPCA summarizes the subject-specific loadings, called principal component scores, by projecting subject curves on the basis of principal components [13]. PC scores can be used in functional regression, so-called Functional principal component regression (FPCR), to assess the effect of fMRI temporal patterns on diagnostic classification.

We propose and implement a use of FPCA on temporal mixing matrices within the context of exact permutation-based conditional logistic regression to analyze risk status for mild cognitive impairment (MCI) in a matched-pairs study. That is, this manuscript considers investigating population variation in brain networks by summarizing temporal mixing matrices using FPCA in conditional logistic regression.

## Materials and Methods

### Sample

The data derived from an ongoing study of Alzheimer's disease progression. This study followed roughly two hundred subjects, such that 100 were at high familial risk for AD while 100 were at low risk. At-risk participants were drawn from families enrolled in an ongoing genetic linkage study, developed at the Johns Hopkins University as part of the NIMH Alzheimer's Disease Genetics Initiative [14], [15]. The at-risk participants all have a parent with Alzheimer's disease, confirmed via autopsy, and at least one additional first degree relative with a clinical diagnosis of probable Alzheimer's disease. All subjects were at least 50 years of age, free of memory complaints scored in the normal range on the Telephone Interview for Cognitive Status (TICS) [16]. None were on treatment for cognitive impairments. Control participants, who were primarily spouses (65%), had no affected parents or first degree relatives and were free of cognitive complaints or treatments. The study was approved by the Johns Hopkins Institutional Review Board and all participants provided written informed consent. Further details on the study can be found in [15].

The study was comprised of three visits, each roughly five years apart. The data for this manuscript come from the second visit. Until the recent completion of the third wave, 13 subjects of the total (at-risk or control) had been declared as mildly cognitive impaired (MCI). These 13 control subjects were matched to cases via age, gender and education. Therefore, our analysis considers 26 subjects, of which 13 showed no evidence of cognitive decline and 13 were declared as MCI. MCI was declared based on the change in their performance on cognitive testing between visits 1 and 2. For each component of each cognitive test, we calculated the mean change in score and standard deviation in the entire sample. Those whose scores declined by at least 2 standard deviations from the group mean change on any of these components were classified as MCI decliners. Three individuals declined on the Wechsler Memory Scale (third edition) Logical Memory Test, five on the Wechsler Memory Scale Verbal Paired Associates Test, two on the Wechsler Memory Scale Visual Reproduction, three on the Bushke Selective Reminding Test, four on the Benton Visual Retention Test, and one on the Clock Drawing task. This group was made up of seven males and six females, had a mean age of 68.9 years (SD = 5.2) and a mean education of 14.7 years (SD = 4.3). Eighty-five percent of this group had no ApoE4 allele, while 15% had one ApoE4 allele. Table 1 summaries the demographic information for both MCI and controls.

**Table 1:** Decliner and control characteristics.

	Decliner (n = 13)	Control (n = 13)	Significant Level
Gender	7 Male, 6 Female	7 Male, 6 Female	
Age	68.9 (5.2)	66.2 (5.5)	P = 0.331
Education	14.7 (4.3)	15.1 (3.4)	P = 0.802
ApoE-4 carriers (%)	15%	25%	P = 0.548

### fMRI Scanning Protocol

Three waves of neuroimaging data collection have been completed. The fMRI data used in the analysis are from the second wave, which is concurrent with the measurements used to declare subjects as having MCI. All fMRI data used were part of a protocol that involved a verbal memory paradigm (see below).

Functional neuroimaging was obtained via a 1.5 T Philips Intera-NT scanner (Philips Medical Systems, Best, The Netherlands) at the F.M. Kirby Functional Imaging Research Center (Kennedy Krieger Institute, Baltimore, MD). Two functional scans were acquired with repetition time (TR) = 1000 ms, echo time (TE) = 39 ms, flip angle (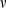) = 90°, field of view (FOV) = 230 mm in the 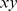 plane and matrix size = 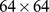 reconstructed to 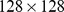. Eighteen slices were acquired with a 4.5 mm thickness and an interslice gap of 0.5 mm, focused on the medial temporal lobe. Hence, for example, much of the anterior portion of the frontal and posterior portion of the occipital lobe were not studied. Every scan was investigated by radiologists and no structural abnormalities were seen in the MRI scans.

### Task procedure

The paradigm was an auditory word-pair association task. It consisted of two 6 minute and 10 second sessions with each session having six sets of three blocks. Three types of blocks were considered: encoding, rest and recall. The encoding phase and the recall phase were 19.5 s long, and the rest phase in between the encoding and recall phases was 17 s long. When in the encoding block, subjects were presented with seven unrelated word pairs. When in the recall block, subjects were presented with the first word of each pair and instructed to silently recall the second. Subjects were asked to keep their eyes open during each block. In the baseline block, which was a rest phase, subjects were presented with an asterisk.

### Data preprocessing

Data pre-processing was conducted using the Statistical Parametric Mapping software (SPM99 Wellcome Department of Imaging Neuroscience, University College, London, UK) running under the MATLAB 6.1 (The Mathworks, Sherborn, MA, USA) programming and run-time environment. SPM99 was used for legacy reasons and to achieve consistency with visit 1 processing. A rigid body motion correction was performed by realigning all the scans from both sessions to the mean image of all the functionals in both sessions. This was followed by re-slicing using a windowed-sinc interpolation. Realigned images were hand checked for motion artifacts. Twelve-parameter nonlinear transformations via SPM99 were used to warp images into standard space (MNI). The template was manually cut to fit each individual scan in order to improve the quality of normalization. Normalized scans were re-sliced to isotropic voxels (2 mm^3^), using trilinear interpolation and spatially smoothed with a full-width at half-maximum (FWHM) Gaussian kernel of 5 mm.

### Independent Component Analysis of fMRI

Independent component analysis is a commonly used method for recovering underlying independent sources from their mixtures, so-called blind source separation. ICA has been frequently utilized on the analysis of functional neuroimaging data since 1998 [7]–[10]. Two key benefits of ICA are its empirical nature and its often considered reasonable underlying generative model. Specifically, it models collected signals as linear weighted combinations of independent sources. Notationally, a noise-free ICA model specifies
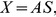
(1)where 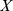 is a 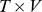 data matrix. Note that, in our application, 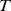 indicates scan while 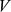 indicates voxels. Rows of 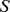 contain the independent components and 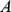 is the linear mixing matrix. Let 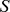 be 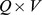 where 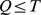 and hence 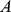 is 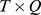. We use parentheses to index matrices so that 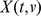 is element 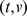 of 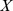 and define 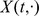 as row 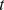 of 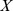 and 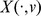 as column 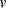. Then, model (1) could be rewritten as 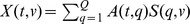 and 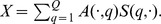


ICA gets its name by assuming that 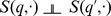 when 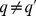 where 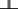 implies statistical independence. However, standard variations of ICA also assumes that 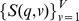 is an iid collection, which we also adopt. As a consequence of these assumptions, 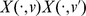 when 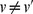; yet note that 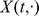 is not necessarily independent of 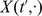.

FastICA is a fixed-point scheme frequently used for independent component estimation (see http://www.cis.hut.fi/projects/ica/fastica/) by iteratively maximizing negative entropy. We use fastICA as our optimization criteria in this manuscript, as it is a popular ICA fitting algorithm, though note that the proposed analysis stream is largely agnostic to this choice. Note further that we use a so-called ‘noise-free’ ICA model. Of course, such assumptions are not realistic for fMRI and hence measurement error and other sources of variation will be absorbed in the estimated time courses and spatial maps. Our simulations, however, show that this does not impact our regression approach. Regardless, we reiterate that one could use a probabilistic ICA method [17] for estimation instead.

### Group independent component analysis

We now consider ICA on groups of subjects and its inferential consequences. There are a wide variety of group ICA approaches for multi-subject fMRI data [18]. We follow standard practice and consider a temporal concatenation approach, so called spatial group ICA [9]. Spatial independent component analysis decomposes fMRI data into spatial maps multiplied by their respective time courses, where the maps are drawn from spatial distributions that are statistically independent [9].

Notationally, let 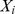 be a 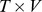 (scan by voxel) matrix for subject 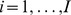. We consider the ICA model 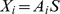, where 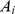 is a 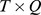 matrix of temporal weightings and 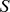 is a 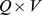 matrix of ICs. Let 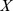 be the 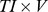 matrix obtained by stacking the 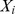 and 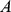 be the 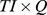 matrix obtained by stacking the 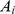; that is, 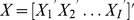 and 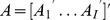. Then, spatial group ICA simply specifies the standard model 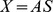, with the only notational difference with the previous section being that 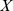 and 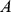 each now have 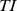 rows rather than 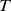. The biological interpretation of group ICA is that fMRI intensities represent BOLD signals across brain networks that are common across subjects. However, how each subject temporally loads these networks can vary. An SVD decomposition (or some other dimension reduction) is still required to force the ICA model to be identified. Hence, the concatenated data matrix is projected onto the left singular vectors, an ICA is performed on the dimension reduced spatial maps, the resulting weight matrices are then projected back into the original data space. If 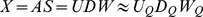 where 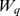 is the submatrix containing the first 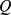 rows of 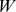, 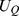 contains the first 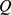 columns of 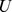 and 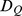 is the upper 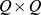 matrix of 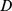. Treating the approximation as if it were an equal sign we obtain that 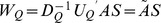. After ICA estimation of 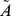, say resulting in 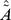, we set 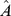 as 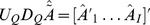.

### Population functional analysis of group ICA

The temporal weight matrices, 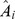, estimate how the common spatial ICs are modulated over scan. The relationship 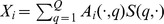 demonstrates how the temporal weight vector 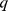, 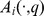, influences the spatial IC, 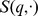. The variable 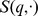 is often interpreted as a brain network. Since network 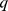 is common across subjects, one can analyze the 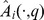 across subjects to investigate inter-group differences in network behavior. We propose generalized functional regression as a tool for such explorations.

Generalized functional regression is a powerful tool to explore the association between functional variables and scalar outcomes, such as binary disease outcomes [11], [19], [20]. Here, we propose the functional variables to be the temporal mixing vectors, 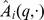. We start with univariate functional analysis. That is, by fixing 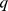 a specific index for the independent component, we only consider one functional regressor at a time, though acknowledge that multivariate regression models are a relatively straightforward extension. Let 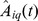 be a function representing the vector 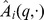. The particulars of how to take densely sampled discrete data and represent it as a continuous function can vary. We use a PCA decomposition and variance matrix smoothing as our method, with details given below.

Assume for each subject 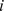, 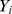 is the scalar outcome, 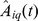 are random functions for fixed 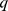. Let 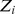 be a vector of nonfunctional covariates, including the intercept. Without loss of generality, we assume that the 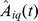 are mean zero stochastic processes (which can be achieved in practice by subtracting the population average function). The functional regression model can be expressed as:
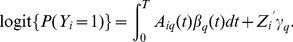
(2)In model (2), the functional parameter 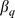 is the main target of inference. It is interpreted as a weighting scheme, which tends to emphasize or de-emphasize components parts of 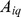. In our context, it relates the temporal mixing matrices to the disease status outcomes. However, a functional expansion of 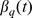 needs to be performed to obtain a finite dimensional parameter for fitting.

### Dimension reduction by functional principal component analysis

We use Functional Principal Component Analysis (FPCA) to calculate the eigenfunctions of 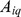 that capture most of the population variability of network-specific temporal mixing. This has the benefit that PCA of group ICA temporal mixing matrices is already a common technique in the neuroimaging literature. Moreover, eigenfunction decompositions simultaneously yield a convenient, data-driven basis for which to decompose model (2) into easily estimated parts, as well as recasts the problem in the terms of the greatest direction of inter-subject variation in the temporal mixing of ICA-based brain networks.

FPCA considers a complex functional regression space by decomposing the covariance operator 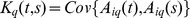. Let the spectral decomposition of this covariance matrix be given by 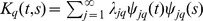 (as noted in [13], [21], via Mercer's theorem). Here 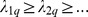 are the ordered eigenvalues and 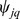 are the associated orthonormal eigenfunctions. The spectral decomposition yields a parsimonious expansion of the subject level functions 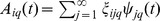, referred to as the Karhunen/Lo
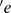
ve (KL) decomposition [22], [23]. Here, 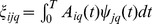 are referred to as the principal component scores. Necessarily, we truncate the decomposition at 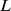 terms (though see [20]) so that 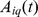 has a finite decomposition expression, 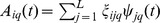. Details about how to select 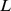 are given in Appendix S1.

The true mixing matrix functions, 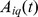, are not observed. Instead we obtain the model-based estimates from the ICA algorithm, 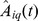. Assume a measurement error model so that 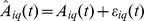, where 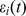 is a white noise process with variance 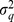. Thus a smoothing step is desirable [24] with details given in Appendix S1.

Once the eigenfunctions, 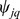, and truncation lag, 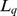, are fixed, the model for the noisy signals can be written as:
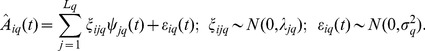
(3)As stated in [19], this is a linear mixed model with random effects 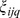 used to model the outcome. A benefit of this model is the ability to use BLUP estimation to estimate 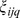.

Since 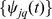 is an orthonormal basis in 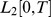, both 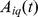 and 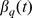 have unique representations as linear combinations of this basis. Thus, equation (2) can be rewritten as:
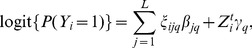
(4)where 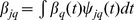. This is nothing other than a standard (non-functional) logistic regression model.

Following the definitions of [19], model (3) is the exposure model and model (4) is the outcome model. In our context, the exposure model considers the temporal mixing matrices from group ICA; the outcome model relates the principal components from the exposure model to a disease status outcome model. In our application, the outcome status is fixed by design and hence we employ the logic of traditional case-control logistic regression [25], [26].

### Functional connectivity

We argue that interaction models are of key interest. Specifically, functional connectivity is biologically meaningful because it is assumed that “memory and other cognitive abilities are the result of the integrated activity in networks of regions, rather than activity in any one region in isolation” [2]. Joel et al [27] argue for the important interpretation of inter- and intra-network connectivity ostensibly measured by the correlation and variance of the temporal mixing matrices respectively. As these matrices have zero mean, we consider their products and squares. Mathematically, ICA-based inter-network functional connectivity is defined as 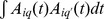 and intra-network functional connectivity is defined as 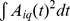 [27].

Joel et al [27] suggested the estimated counterparts of the inter- and intra-network connectivity as predictors of disease. We can easily incorporate these summaries into our framework with the models
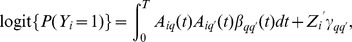
and
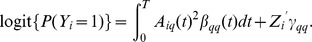
These models are simple realizations of our existing functional model with functional regressors 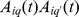 and 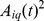, respectively. The only slight complication is that an additive functional measurement error model would likely not hold for 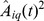. Notice that if the coefficient functions are estimated to be constants, these models simply regress the outcome on the measures of inter and intra-network connectivity suggested by [27].

### Hypothesis testing

Finally, we mention small number of subjects is the norm in this area. Therefore, for testing effects, such as the hypothesis 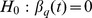, we suggest exact logistic regression. Specifically, under the null hypothesis of no functional effect, 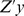 are minimal sufficient statistics for 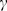, where 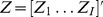 and 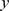 is the observed version of 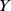. Therefore, the conditional distribution 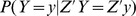 is parameter free. In our cases, if 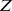 contains only an intercept, then the null distribution is the permutation distribution of outcomes. If 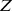 contains pair-specific indicators for matched pairs data, then the null conditional distribution is the distribution obtained by permuting the outcomes among discordant pairs. It should also be noted that if 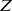 contains non-categorical covariates, or too many categorical covariates, especially interaction terms, the conditional distribution can be uninformative and the resulting P-values can be excessively conservative. Agresti [28] has more details about variations on conditional logistic regression.

### Related work

Beckmann and Smith [29] proposed a tensor PICA model, which factors the group data as a trilinear combination of three outer products, representing group spatial maps and time courses but subject-specific loadings. The tensor PICA is a simplified version of our model in the sense that, by assuming common time courses, they only retain one eigenvector in the PCA stage. We can check the validity of their model by calculating the ratio of the largest eigenvalue and the sum of all the eigenvalues. Their approach may not work well if the temporal dynamics are different across subjects, such as in a resting state study [18]. Our method allows heterogeneity in time courses and hence may be more robust.

An alternative two-stage decomposition for the analysis of fMRI data was proposed by Caffo et al [30]. Their approach first used singular value decomposition (SVD) to obtain subject-specific eigenimages (spatial maps) and eigenvariates (time series). Then the collections of eigenimages and eigenvariates were decomposed to form population-level brain networks and time series. Subject-level data were projected onto these population eigenvectors to obtain subject-specific loadings and those loadings can be used in generalized functional regression. One potential weakness of their approach is the ignorance of variance ordering of subject-specific eigenvectors in the population analysis. Comparatively, the components in ICA are not ordered, so the problem is avoided. In addition, the SVD forces orthogonal eigenimages and eigenvariates, which may or may not reflect actual biology. In principle, the relevant information content of ICA-based regressors is equivalent to SVD-based regressors for FPCA. However, the ICA-based regressors tend to be more interpretable by not requiring orthogonality of the time courses.

In addition, Zhu et al. [31] proposed a functional analysis pipeline, called Functional Analysis of Diffusion Tensor Tract Statistics (FADTTS), for delineating the association between multiple diffusion properties along major white matter fiber bundles with a set of covariates of interest. Though FADTTS was proposed in the context of diffusion tensor imaging (DTI), it can be applied to the second stage of our analyses as an alternative way. Specifically, one can apply FADTTS to the time courses 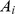 as response and include the diagnostic status as covariates. Hypothesis testing can be done to identify the time points where the two groups differ from each other.

## Results

### Independent component analysis of fMRI data in the AD study

We apply our methods to the Alzheimer's disease risk study. The fMRI data contain 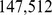 non-background voxels measured at 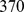 time points, with a TR of 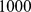 ms, for each subject. A group data matrix is generated by concatenating 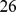 subjects' fMRI data in the temporal domain. The aggregated matrix has dimension 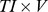, where 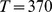, 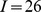, and 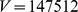.

In the practical application of ICA, identifying the number of independent components is an important step. For fMRI, the number of informative components is often assumed to be less than the spatial or temporal dimension; further the mathematics mandate that the number of ICs be less than the smaller of 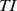 and 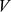 (typically 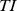). This manuscript adopts a simple approach to estimate the number of components based on the eigenvalues of the covariance matrix.

Note that 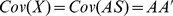. Making the small allowance of adding a nugget variance adds a diagonal constant to this matrix. There are 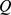 non-zero eigenvalues to this matrix, with the rest being equal to the nugget variance. The scree plot shows that eigenvalues decay to a constant roughly at 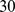. So we apply ICA on the group data matrix specifying there are 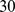 independent components. The retention of all 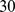 components in subsequent modeling could potentially have issues of variance inflation. Moreover, we stipulate that the choice is admittedly ad hoc. We do not address this further in this manuscript, though emphasize the importance of performing sensitivity analysis to the number of retained eigenvalues. In addition, we do note success in using penalties terms in functional regression that are insensitive to the choice of the number of components [20].

### Functional regression on mixing matrix

In the second stage, PCA is performed on the time courses for each independent component acquired from ICA. We then apply functional logistic regression on the scores derived from PCA, accounting for case-control matching. There are 30 spatial maps specified from ICA, so we conduct 30 regressions, with each regression on PC scores summarized from time courses associated with one specific spatial map. In each regression, there are up to six PC scores (i.e. the predictors), where the number of PC scores are estimated using criterion stated in Appendix S1.

In our application, there are 26 subjects, with 13 MCI subjects and 13 matched controls. As discussed earlier, the null conditional logistic regression distribution permutes outcomes among discordant pairs. We then obtain the likelihood ratio test (LRT) statistic under each permutation, and calculate the Monte Carlo estimated probability that the LRT statistic exceeds the actual observed LRT statistic. The null permutation distribution in this case (matched binary pairs) is equivalent to randomly permuting case status among matched pairs. No adjustment for multiplicity is made, as our results are exploratory in nature.

**Figure 1:**
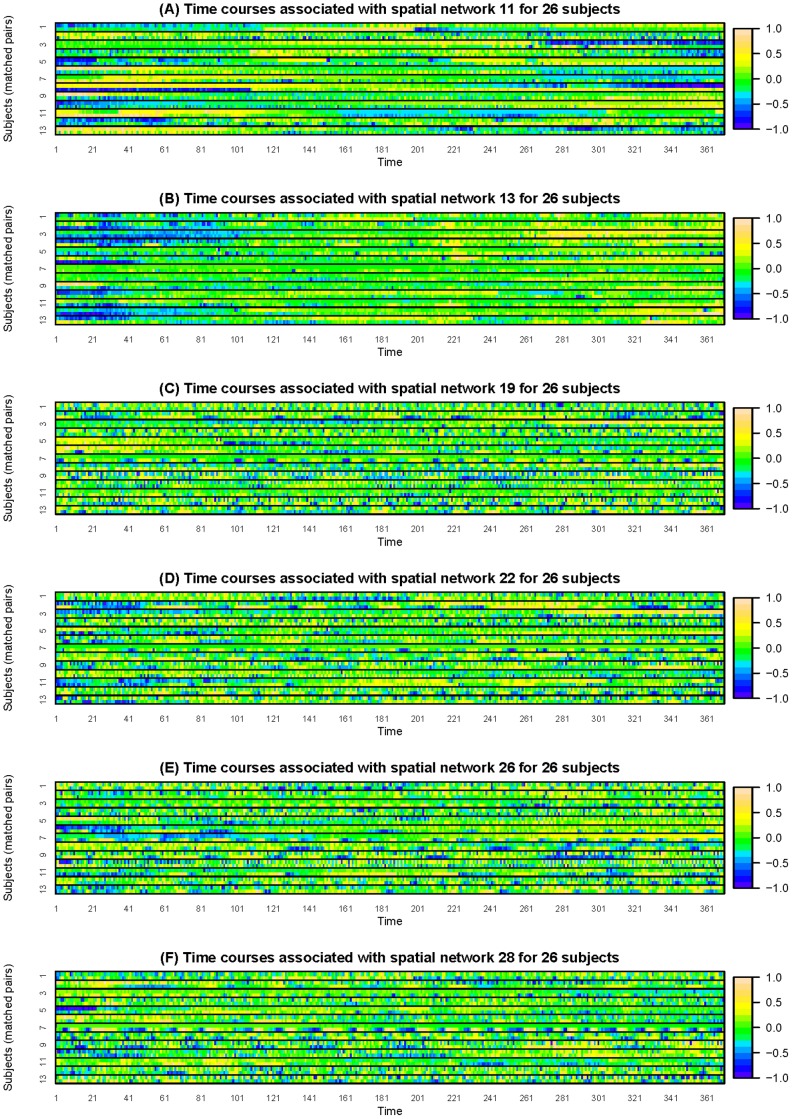
Plots (A)∼(F) are heatmaps for time courses modulating spatial maps 11, 13, 19, 22, 26, 28 respectively. The subjects are grouped by matched pairs.

**Figure 2:**
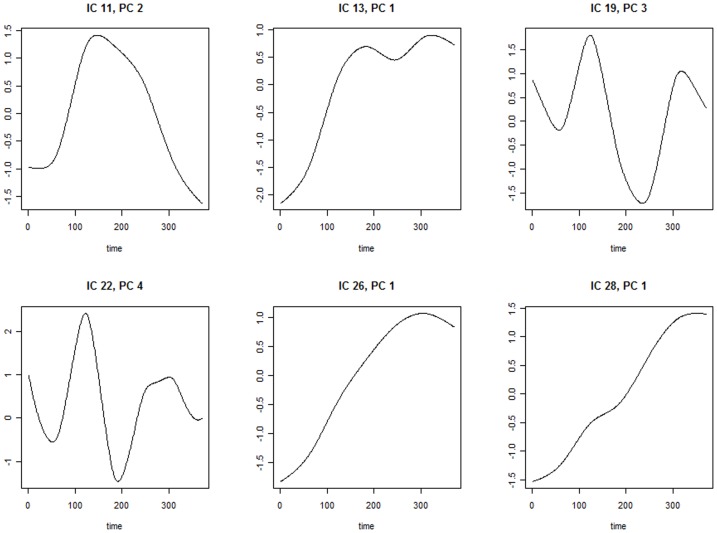
Plots of eigenfunctions associated with the significant predictors.

Table 2 summarizes the regression results. Of the 30 regression, 6 of them show significant results. Significant predictors are shown in table 2. Figure 1 exhibits the time courses that are associated with odds of MCI for all 13 matched pairs of subjects. To avoid overlap of the trajectories among subjects, heatmaps of the time courses are provided instead of spaghetti plots [32]. The heatmaps better visualize trends and patterns in the network-specific temporal mixing matrices. Figure 2 exhibits the eigenfunctions of time courses in Figure 1. Three predictors are negatively associated with odds of MCI, including the first PC score of time courses modulating spatial map 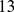, the third PC score of time courses modulating spatial map 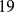 and the first PC score of time courses modulating spatial map 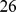. For example, a subject with one unit higher in the first PC of time courses modulating spatial map 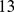 has an estimated 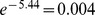 times the odds of MCI. After standardization, one standard deviation increase in the first PC score of time courses modulating spatial map 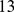 is associated with an estimated odds ratio of 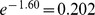. Three predictors are positively associated with MCI, including the second PC score of time courses modulating spatial map 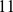, the fourth PC score of time courses modulating spatial map 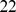 and the first PC score of time courses modulating spatial map 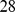.

**Table 2:** AD study: univariate regression analysis results.

	Estimate	SE	LRT	Permutation Test
IC 11, PC 2	9.19	5.59	p = 0.028	p = 0.039
IC 13, PC 1	−5.44	3.40	p = 0.050	p = 0.071
IC 19, PC 3	−25.00	14.60	p = 0.016	p = 0.023
IC 22, PC 4	20.40	9.98	p = 0.009	p = 0.011
IC 26, PC 1	−3.70	2.13	p = 0.030	p = 0.039
IC 28, PC 1	7.82	4.90	p = 0.024	p = 0.043

**Figure 3:**
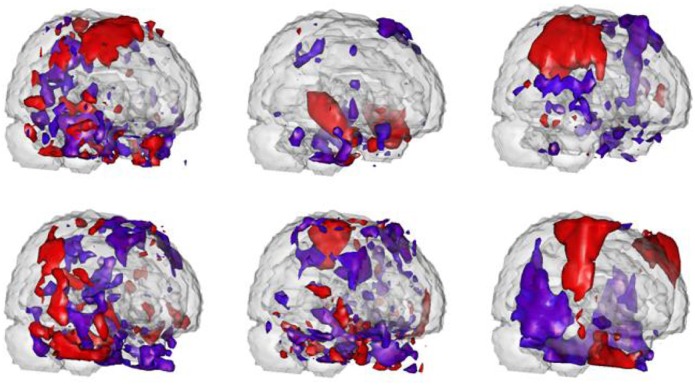
Three-D rendering of thresholded spatial maps associated with the significant predictors. Red areas load positively while blue areas load negatively. The figures from the upper left to the upper right are spatial maps of IC 11, 13 and 19 respectively. The figures from the lower left to the lower right are spatial maps of IC 22, 26 and 28 respectively.

**Figure 4:**
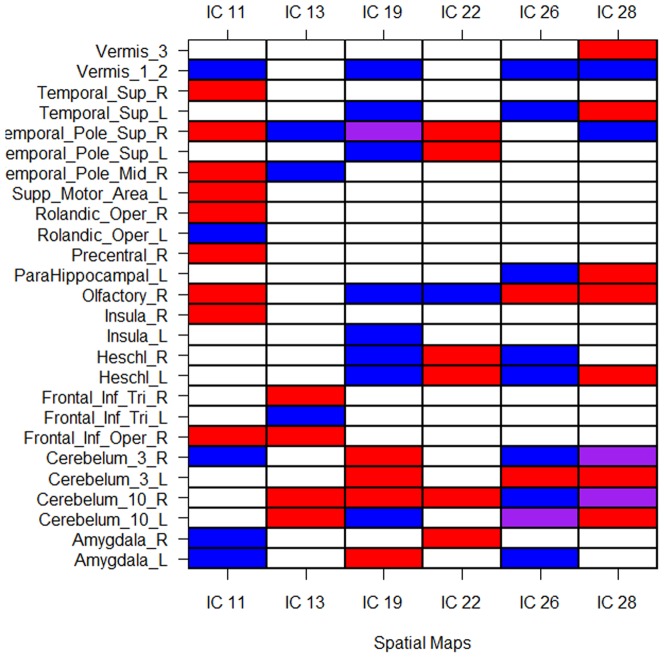
Regions with over 20% overlap with the specified spatial maps. Red areas load positively, blue negatively, yellow have partial volumes loading positively and negatively. Abbreviations: Amyg. = Amygdala, Cer. = Cerebellum, Fr. = Frontal, Hippo = hippocampus, Inf. = Inferior, Ins. = Insula, L. = Left, Olf. = Olfactory, Op. = Opercular part, Pal. = pallium, PHG = Para-Hippocampal Gyrus, Put. = putamen, R. = Right, Sup. = Superior, Temp. = Temporal, Tri. = triangularis.

The spatial maps 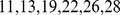 are regions of interest, that is, their corresponding PC scores are significant predictors in the functional regression model. Figure 3 exhibits three-D rendering of these spatial maps. Figure 4 displays brain regions that have over 20% overlap with the identified spatial maps, based on the anatomical parcellation given in [33]. Because of the narrow imaging area, the spatial independent components overlap heavily on temporal regions. The most significant predictor is IC number 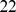. This map is primarily located in the temporal poles, olfactory areas and Heschl regions. This mirrors results found in [30], who studied at-risk subjects versus controls rather than subjects with evidence of MCI and further focused on the singular value decomposition rather than ICA. In addition, a less significant region, 13, overlaps with the hippocampus, the primary brain region of interest in the study of AD. Note specifically that most of these spatial maps intersect with the olfactory areas, which have been hypothesized to be associated with neurodegenerative disorders and Alzheimer's disease in particular [34]. Looking across regions, the temporal poles, Heschl regions, cerebellum, amygdala and limbic olfactory areas are widely implicated across ICs. However, the results are non-specific.

To study the association between inter-network functional connectivity and MCI, we apply conditional logistic regression on the product of time courses associated with two different spatial maps. The results are listed in Table 3. After standardization, one standard deviation increase in the second PC score of the integrated activity of network 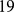 and 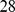 is associated with an odds ratio 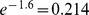; one standard deviation increase in the second PC score of the integrated activity of network 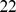 and 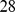 is associated with an odds ratio 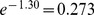; one standard deviation increase in the third PC score of the integrated activity within network 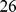 is associated with an odds ratio 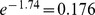. Based on anatomical parcellation, specific AD-related network interactions include connectivity between cerebellum and vermis, frontal temporal lobe and cerebellum, frontal temporal lobe and vermis, cerebellum and Heschl gyrus.

**Table 3:** AD study: regression results using the functional connectivity as the predictors.

	Estimate	SE	LRT	Permutation Test
IC 19 and 28^a^, PC 2	−11.60	7.51	p = 0.007	p = 0.015
IC 22 and 28^b^, PC 2	−36.80	28.40	p = 0.037	p = 0.050
IC 26 and 26^c^, PC 3	−75.9	43.7	p = 0.026	p = 0.031

a The between network connectivity of spatial maps 19 and 28.

b The between network connectivity of spatial maps 22 and 28.

c The within network connectivity of spatial map 26.

### Limitation

The style of analysis is exploratory and empirical. Our prior hypothesis involves associations with paradigm-related limbic structures. Evidence of such effects is seen to this end. However, the results are non-specific. These results must be viewed with caution given the small number of MCI cases available for study.

In addition, in the process of considering multiple components, multiplicity concerns are an issue. Coupled with the fact that the motivation for this study is from a study of the same data, the results must be interpreted with care. External validation on new data sets is a goal for future research.

## Discussion

In our application, we found that our approach discovered potentially interesting relationships between estimated brain networks and disease states. In the AD study, our results are similar to [30], though considering only 26 carefully matched subjects to nearly 200 in that study. Moreover, our investigation considered subjects with MCI, not subjects at-risk of disease. Being post-hoc analyses, these results must be viewed with some skepticism. It would be of use to validate findings with another study employing a similar paradigm.

We comment particularly on the use of functional regression in the resting state studies, a mainstay of modern fMRI research. In principle, resting state scans are not temporally aligned across subjects. Therefore, one must be careful in any technique that assumes temporal alignment, as our functional regression models do. Note, however, a constant 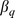 function results in the use of standard resting state ICA metrics as predictors in the logistic regression model.

Besides, one difficulty in analyzing fMRI group data is its high dimensionality. Our approach includes two steps. In the first stage, we attempt to decompose a group matrix with dimension 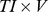. In the second stage, we perform functional PCA regression on the time courses matrix with dimension 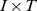. Hence, the computational time is mostly spent on the first stage group ICA. Thus our approach allows for thorough investigation of second stage models and covariates with only modest computing times. For studies with large number of subjects, the 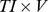 matrix may be too large in both dimensions to admit group ICA without dimensionality reduction. By conducting data reduction in the temporal domain, we could reduce one dimension of the large matrix to make it computationally practicable [9], [29]. However, given the increasing scope of fMRI data, novel implementations of group ICA for large numbers of subjects remains needed.

Finally, we comment that this manuscript addresses decomposition methods to evaluate cross-sectional variation in brain networks. However, the structure can be extended to hierarchical models. Longitudinal functional imaging studies are becoming increasingly common. For example, subjects may have fMRI records at multiple visits. Our two-stage method can be generalized for multi-level data very easily, provided longitudinal functional regression models, an active area of research [13], [35].

## Supporting Information

Appendix S1 (PDF)
