# Supplementary material for: Analysis of Group ICA-Based Connectivity Measures from fMRI: Application to Alzheimer's Disease
Source: PLoS One. 2012 Nov 30;7(11):e49340. doi: 10.1371/journal.pone.0049340 (PMC3511486; doi:10.1371/journal.pone.0049340)
Supplement: Appendix S1 — (PDF) [file pone.0049340.s001.pdf]

# Supporting Information Legends

### Estimating Number of Principal Components

The spectral decomposition yields a parsimonious expansion of the subject level functions $A_{iq}(t) = \sum_{j=1}^{\infty} \xi_{ijq}\psi_{jq}(t)$. Necessarily, we truncate the decomposition at $L$ terms so that $A_{iq}(t)$ has a finite decomposition expression, $A_{iq}(t) = \sum_{j=1}^{L} \xi_{ijq}\psi_{jq}(t)$.

We follow the approach proposed by [?] to estimate $L$ based on proportion of variance explained. Let $P_1$ and $P_2$ be two thresholds, and define

$$L_q = \min\{k : \sum_{j=1}^{k} \lambda_{jq} / \sum_{j'=1}^{\infty} \lambda_{j'q} \geq P_1, \lambda_{kq} < P_2\}.$$

Here, $P_1$ is a threshold on the cumulative explained variance while $P_2$ is a threshold on the individual explained variance. In this manuscript, we choose $P_1 = 0.95$ and $P_2 = 0.02$. These choices work well in our simulations and application. However, they should be carefully tuned in other settings, perhaps using simulations.

### Variance Matrix Smoothing

Instead of the true mixing matrix functions, $A_{iq}(t)$, we obtain the model-based estimates from the ICA algorithm, $\hat{A}_{iq}(t)$. Assume a measurement error model so that $\hat{A}_{iq}(t) = A_{iq}(t) + \epsilon_{iq}(t)$, where $\epsilon_i(t)$ is a white noise process with variance $\sigma_q^2$. Thus a smoothing step is desirable.

Under the assumed model $\hat{A}_{iq}(t) = A_{iq}(t) + \epsilon_{iq}(t)$, the covariance operator for the observed data is $K_q^W(s,t) = K_q(s,t) + \sigma_q^2 \delta_{ts}$, where $K_q^W(s,t) = \mathrm{Cov}\{\hat{A}_{iq}(s), \hat{A}_{iq}(t)\}$ and $\delta_{ts} = 1$ if $t = s$ and 0 otherwise [?]. This equation reveals that the diagonal elements of $K_q^W(s,t)$ includes the nugget measurement error. A simple and natural solution is to drop the diagonal elements and smooth the covariance matrix. We use the standard (moment based) estimate $\hat{K}_q^W(s,t)$ from the observed data, $\sum_{i=1}^{I} \hat{A}_{iq}(t)\hat{A}_{iq}(s)/I$ and then estimate $\hat{K}^A(s,t)$ by smoothing the estimate for $s \neq t$ [?, ?]. The eigenvalues, $\lambda_{jq}$, and eigenfunctions, $\psi_{jq}$ can then be derived from this estimated covariance matrix.
